# Dietary Supplementation of ε-Polylysine Beneficially Affects Ileal Microbiota Structure and Function in Ningxiang Pigs

**DOI:** 10.3389/fmicb.2020.544097

**Published:** 2020-11-16

**Authors:** Xuelei Zhang, Zhenping Hou, Baoyang Xu, Chunlin Xie, Zhichang Wang, Xia Yu, Duanqin Wu, Xianghua Yan, Qiuzhong Dai

**Affiliations:** ^1^Institute of Bast Fiber Crops, Chinese Academy of Agricultural Sciences, Changsha, China; ^2^Department of Animal Nutrition and Feed Science, College of Animal Science and Technology, Huazhong Agricultural University, Wuhan, China

**Keywords:** ε-polylysine, Ningxiang pig, ileum, microbiota, nutrient digestibility, metabolism

## Abstract

Intestinal microbiota plays an important role in the health of animals. However, little is known about the gut microbiota in Ningxiang pigs. Thus, we investigated how dietary supplementation with different ε-polylysine concentrations (0, 20, 40, 80, and 160 ppm) affected the ileal microbiota in Ningxiang pigs using a replicated 5 × 5 Latin square method. Each experimental period included 10 days for diet adaptation, 3 days for feces collection and 2 days for digesta collection. The ileal contents were collected and used for sequencing of the V3–V4 hypervariable region of the 16S rRNA gene. The results revealed that ε-polylysine significantly decreased the digestibility of crude protein and crude fiber, as well as the utilization of metabolizable energy (*P* < 0.05). The relative abundances of 19 bacterial genera significantly increased, while those of 26 genera significantly decreased (*P* < 0.05). In addition, ε-polylysine increased the abundance of some bacteria (e.g., *Faecalibacterium*, *Bifidobacterium*, and lactic acid bacteria) and inhibited some other bacteria (e.g., *Micrococcaceae*, *Acinetobacter*, *Anaerococcus*, *Peptoniphilus*, *Dehalobacterium*, *Finegoldia*, *Treponema*, and *Brevundimonas*). Furthermore, based on the 16S rRNA gene data and data from the precalculated GreenGenes database, bacterial communities in the ileal contents exhibited enhanced functional maturation, including changes in the metabolism of carbohydrates, amino acids (e.g., alanine, lysine, tryptophan, cysteine, and methionine), cofactors, and vitamins (e.g., biotin, thiamine, and folate), as well as in the activity of the insulin signaling pathway. This study suggests that ε-polylysine may influence the utilization of feed nutrients by Ningxiang pigs, including proteins, lipids, metabolizable energy, and fiber, by regulating the gut microbiota.

## Introduction

Intestinal microbiota is a key to many aspects of nutrition and health, including the immune system (Zhang et al., 2015; Hu et al., 2018), neurobehavioral traits (Chu et al., 2019), digestion, and metabolism (Sonnenburg and Sonnenburg, 2014; Rothschild et al., 2018), as microorganisms enable dietary fiber fermentation and affect energy metabolism (Valdes et al., 2018). An interactive relationship exists between intestinal microbiota and nutrition, wherein intestinal microbiota affects the digestion/absorption of nutrients and diet/nutrition affects the diversity of the gut microbiota (Flint et al., 2012; Oriach et al., 2016; Valdes et al., 2018).

The nutritional food additive ε-polylysine achieves its antibacterial effects by increasing the permeability of the cell membrane. ε-Polylysine exhibits a broad spectrum of bacteriostatic properties and affects gram-positive bacteria, fungi, and some viruses. Studies in the field of food and nutrition have demonstrated that ε-polylysine exerts antimicrobial activity against *Listeria monocytogenes, Escherichia coli* O157:H7, and *Salmonella* Typhimurium (Geornaras and Sofos, 2005; Geornaras et al., 2007; Chang et al., 2010; Zhou et al., 2011). Antibacterial tests indicated that the membrane fraction between 2 and 5 kDa exhibited the highest antibacterial activity compared with that of other fractions against test strains of *Staphylococcus aureus, Micrococcus luteus*, *Bacillus subtilis*, *E. coli*, and *Shigella* spp. (Jia et al., 2010). Furthermore, ε-polylysine is easily adsorbed by DNA owing to its negative charge, which makes ε-polylysine suitable for the treatment of liver diseases. Polymerized protein-carrying biological macromolecules of ε-polylysine can effectively treat hepatitis virus infection (Sospedra et al., 1999a, b). One study reported that when antimicrobial ε-polylysine was incorporated into food, it transiently altered the gut microbial communities, as well as their predicted functions, in mice, which indicates a dynamic, yet resilient microbiome that adapts to microbial-active dietary components (You et al., 2017). ε-Polylysine can decrease triacylglycerols by inhibiting pancreatic lipase activity (Kido et al., 2003; Tsujita et al., 2006). Furthermore, Hosomi et al. (2015) reported that hepatic acetyl-coenzyme A carboxylase and glucose-6-phosphate dehydrogenase, two key enzymes of fatty acid biosynthesis, were enhanced in rats that were fed a diet with ε-polylysine.

The present study was performed on the Ningxiang pig (also known as the Caochong or Liusha River pig), a famous native pig breeds in the Hunan province in China, which possesses unique hereditary properties, including a high reproduction rate and good adaptability (Xing et al., 2009). At present, research on ε-polylysine is mainly concentrated in the fields of food and medicine, and ε-polylysine is considered to play an important role as a nutritional antiseptic and antibacterial agent. However, its application in poultry and livestock nutrition has not yet been reported; in particular, its effects on animal gut microbiota remain to be explored. Thus, we aimed to assess whether dietary supplementation with ε-polylysine could regulate the digestion of nutrients and beneficially affect the microbiota structure and function in the ileum of Ningxiang pigs.

## Materials and Methods

### Ethics Statement

All experimental protocols were carried out with the approval of the Institute of Bast Fiber Crops of the Chinese Academy of Agricultural Sciences, Hunan province, China.

### Animals and Sample Collection

Five adult Ningxiang pigs were surgically fitted, between the ileum and cecum, with a T-shaped cranial cannula of approximately 15 cm in length, made of polyvinylchloride plastisol, according to the procedures suggested by Sauer et al. (1977). After a 1-month convalescence period, the pigs were tested in separate stainless-steel metabolic cages. The intubation did not appear to affect the growth of the animals, as indicated by the weight gain and feeding efficiency. The trial was conducted as a replicated 5 × 5 Latin square design with pigs and feeding periods as blocking factors, which is shown in Table 1. Each experimental period lasted 15 days, including 10 days of diet adaptation and intake adjustment, 3 days of feces collection and 2 days for the collection of digesta from the ileum (12 h/day). All the pigs were fed a basal diet supplemented with 0, 20, 40, 80, or 160 ppm ε-polylysine. The composition of the basal diet is shown in Table 2. Feces were collected and weighed every day. Sulfuric acid (H_2_SO_4_) added was 10% of the weight of fresh feces to protect samples from decaying. Feces were collected and mixed well in a plastic bag, and 10% of the mixed feces was kept for analysis. The collected feces and feed samples were dried in a forced-air drying oven at 65°C and then ground to pass through a 40-mesh sieve for analysis. The ileal content samples were collected from the ileal fistula of the pigs. The pH of the ileal contents was measured using a pH meter (model S210 SevenCompact^TM^; Mettler Toledo Instruments Co., Ltd., Shanghai, China) immediately after sampling. Afterward, all ileal content samples were stored at −80°C until further use.

**TABLE 1:** 5 × 5 Latin square design.

Pigs Periods	Pig 1	Pig 2	Pig 3	Pig 4	Pig 5
Section 1	0 ppm	20 ppm	40 ppm	80 ppm	160 ppm
Section 2	20 ppm	40 ppm	80 ppm	160 ppm	0 ppm
Section 3	40 ppm	80 ppm	160 ppm	0 ppm	20 ppm
Section 4	80 ppm	160 ppm	0 ppm	20 ppm	40 ppm
Section 5	160 ppm	0 ppm	20 ppm	40 ppm	80 ppm

**TABLE 2:** Compositions and nutrient levels of the experimental diets (air-dry basis%).

Items	Content (Basic diet)
**Ingredient (%)**	
Corn	39.50
Wheat bran	20.00
Soybean meal (43%)	19.50
Corn starch	8.50
Rice husk powder	5.00
Soybean oil	2.50
Stone powder	1.80
Premix^1^	3.20
Total	100.00
**Nutrient levels (%)**	
DE/(kcal/kg)^2^	3108.80
CP^3^	14.53
CF^3^	6.32
EE^3^	4.16
Ca^3^	0.73
Total P^3^	0.46
Non-phytate P^3^	0.17
Lys^3^	0.79
Met^3^	0.24
Thr^3^	0.56

*^1^The premix provided the following per kg of the diet: Fe 66 mg, Cu 6 mg, Zn 54 mg, Mn 15 mg, I 0.24 mg, Se 0.18 mg, VA 18 000 IU, VD 35 000 IU, VE 35 IU, VK 5 mg, VB_1_ 5 mg, VB_2_ 10 mg, VB_12_ 35 μg, nicotinic acid 40 mg, pantothenic acid 20 mg, folic acid 1.5 mg. ^2^Calculated values. ^3^Analyzed values.*

### Nutrient Digestibility Measurements

Samples of feeds and feces were analyzed for crude protein (CP; AOAC official method 990.03), crude fiber (CF; AOAC official method 978.10), and metabolizable energy (ME). CP was determined by the Kjeldahl method using an auto Kjeldahl system (Kjeltec 2300 Autoanalyzer, Foss Tecator AB, Höganäs, Sweden) after acid digestion. ME was determined using a calorimeter (5E-C5508; Kaiyuan, Changsha, China).

### DNA Extraction

DNA was extracted from the samples using the E.Z.N.A.^®^ stool DNA kit (D4015; Omega, Inc., United States) according to the manufacturer’s instructions. DNA was quantified using an ND-2000C spectrophotometer (NanoDrop Technologies, United States), and its purity was confirmed by agarose gel electrophoresis. The DNA samples were stored at −80°C for polymerase chain reaction (PCR).

### PCR Amplification and 16S rDNA Sequencing

The V3–V4 region of the bacterial 16S rRNA gene was amplified with slightly modified versions of the 338F forward (5′-ACTCCTACGGGAGGCAGCAG-3′) and 806R reverse (5′-GGACTACHVGGGTWTCTAAT-3′) primers (Fadrosh et al., 2014). The 5′ ends of the primers were tagged with specific barcode per sample and sequencing universal primers. The reaction mixture (25 μL) for PCR amplification contained 25 ng of template DNA, 12.5 μL of PCR premix, 2.5 μL of each primer, and PCR-grade water to adjust the volume (Caporaso et al., 2011). The PCR cycling conditions were as follows: initial denaturation at 98°C for 30 s, followed by 35 cycles of denaturation at 98°C for 10 s, annealing at 54°C/52°C for 30 s, and elongation at 72°C for 45 s, with a final extension at 72°C for 10 min. The PCR products were confirmed by 2% agarose gel electrophoresis. Ultrapure water was used as a negative control to exclude the possibility of false-positive results. The PCR products were purified using AMPure XT beads (Beckman Coulter Genomics, Danvers, MA, United States) and quantified using Qubit (Invitrogen, United States). Amplicon pools were used for sequencing, and the size and number of amplicon libraries were evaluated using an Agilent 2100 bioanalyzer (Agilent, United States) and an Illumina library quantification kit (Kapa Biosciences, Woburn, MA, United States). PhiX control libraries (v3) (Illumina) were merged with amplicon libraries (expected at 30%).

### Sequence Processing and Bioinformatics Analysis

Samples were sequenced on an Illumina MiSeq platform. Based on their unique barcodes, truncated paired-end reads were assigned to samples and merged using FLASH (Magoč and Salzberg, 2011). Quality filtering of the raw tags was performed to obtain high-quality clean tags using fqtrim (v0.94). The procedure used for filtering sequence reads was as follows: (1) barcodes and joint sequence were removed from reads; (2) paired-end reads were combined into a longer tag; (3) a window quality scan was performed on reads, with a default scan window of 100 bp; when the average quality value in the window was lower than 20, the part of the read from the beginning of the window to the 3′ end was cut off; (4) sequences less than 100 bp in length after truncation were removed; (5) sequences with more than 5% of Ns after truncation were removed; (6)Chimera sequences were filtered using the Vsearch (Rognes et al., 2016) software (v2.3.4). Sequences with ≥97% similarity were assigned to the same operational taxonomic units (OTUs) using Vsearch (v2.3.4). Subsequently, representative sequences for each OTU were chosen, and taxonomic data were assigned to each representative sequence using the Ribosomal Database Project classifier. To analyze the dominant species in different groups and to study phylogenetic relationships of different OTUs, multiple sequence alignment was conducted using the MAFFT software (v7.310). The abundance of each OTU was normalized relative to the sample with the fewest sequences. Alpha diversity was used to analyze the complexity of species in a sample by applying the Chao1, Shannon, and Simpson indices, which were calculated using QIIME (version 1.8.0). Beta diversity was used to evaluate the complexity of species among the samples and was calculated using principal coordinates analysis (PCoA) and cluster analysis in the QIIME software (version 1.8.0).

### Functional Profile Analysis of Bacterial Communities Using PICRUSt

Based on the 16S rRNA gene data and the precalculated GreenGenes (v13.5) database (Langille et al., 2013), PICRUSt (v1.1.0) was used to predict the abundances of Kyoto Encyclopedia of Genes and Genomes (KEGG) orthologs and KEGG pathways in the bacterial communities. Functional differences among the samples were compared using the STAMP software (Parks et al., 2014).

### Statistical Analysis

The nutrient digestibility data were analyzed using the general linear model procedure in SAS (version 9.2; SAS Institute, Inc., Cary, NC, United States). Tukey’s contrasts were used for *post hoc* comparisons of the means. *P* < 0.01 was considered highly significant, and *P* < 0.05 was considered significant. Statistical analyses of the 16S rRNA gene data were carried out using the GraphPad Prism 8.0 (GraphPad Software, San Diego, CA, United States), R (v3.0.3), and STAMP software. Statistical comparisons of weighted UniFrac distances among the groups were performed by analysis of similarities. One-way analysis of variance was used for the comparison of alpha diversity between groups. After statistical comparison of the taxa, we used the Benjamini–Hochberg correction to control the false discovery rate using the package “p.adjust” in R. The STAMP software with false discovery rate correction was applied to detect the differentially abundant KEGG pathways between groups. *P* (corrected) <0.05 was considered to indicate statistical significance. Statistical analysis of the taxonomy of the ileal contents among the five groups was conducted using wilcox.test in R. Differences were considered significant at *P* < 0.05. Heatmap diagrams and other plots were created in the R environment (v3.1.2). The relationship between intestinal microorganisms and the utilization of nutrients was calculated using the Pearson product correlation. Statistical and correlation analyses were performed using GraphPad Prism 8.0 (GraphPad Software).

## Results

### Apparent Nutrient Digestibility and pH of the Ileal Contents in Ningxiang Pigs

The effects of dietary supplementation with ε-polylysine on nutrient digestibility are presented in Table 3. The average daily feed intake (ADFI) was not influenced by ε-polylysine (*P* > 0.05). However, significant differences were observed in nutrient digestibility. The CP digestibility and ME utilization were significantly higher in the control group than in the 40, 80, and 160 ppm ε-polylysine experimental groups (*P* = 0.036 and *P* = 0.028, respectively). The CF digestibility was significantly higher in the control group than in the 40 and 80 ppm ε-polylysine groups (*P* = 0.040). However, the pH values of the ileal contents exhibited no significant differences (*P* > 0.05).

**TABLE 3:** Effects of dietary ε-polylysine supplemental level on nutrition digestibility (%) of Ningxiang pigs.

Items	Treatment group					Statistics	
	Control	20 ppm	40 ppm	80 ppm	160 ppm	SEM	*P-*value
ADFI/kg	2.58	2.41	2.46	2.45	2.49	0.052	0.062
CP digestibility/%	89.93^a^	89.23^ab^	87.55^b^	85.08^b^	87.79^b^	0.541	0.036
CF digestibility/%	56.66^a^	47.03^ab^	37.51^b^	43.24^b^	47.29^ab^	2.020	0.040
ME utilization/%	86.72^a^	84.82^ab^	81.41^b^	80.68^b^	82.09^b^	0.739	0.028
pH of ileum contents	7.52	7.66	7.40	7.49	7.18	0.072	0.359

*Data are expressed as MEAN with STDEV; Means with different letters within a column differ (P < 0.05); n = 5 per treatment. Treatment Control group, 0 ppm ε-polylysine; Experiment groups: 20 ppm, 40 ppm, 80 ppm, 160 ppm ε-polylysine. ADFI, average daily feed intake; CP, crude protein; CF, crude fiber; ME, metabolizable energy.*

### Characterization of Sequencing Data and Ileal Microbiota in Different Groups

For the five pig groups, a total of 38,170, 36,354, 31,464, 41,236, and 37,242 raw sequences were obtained, of which 76.7, 79.5, 86.2, 83.4, and 78.6% valid sequences, respectively, remained after filtering out chimeras, removing low-quality sequences, and splitting each file in four. Among the high-quality sequences, approximately 58.9% were between 300 and 400 bp, 39.4% were between 400 and 500 bp, and the rest were shorter than 300 bp (Supplementary Figure S1). The Good’s coverage for all samples was >99.5%, which indicated that the sequencing data were reliable (Supplementary Figure S2). Based on the 97% sequence similarity, the sequences of the V3–V4 region were assigned to a total of 3790 bacterial OTUs. The taxonomic analysis revealed a total of 17 bacterial phyla, 32 classes, 54 orders, 116 families, 344 genera, and 672 species.

**FIGURE 1:**
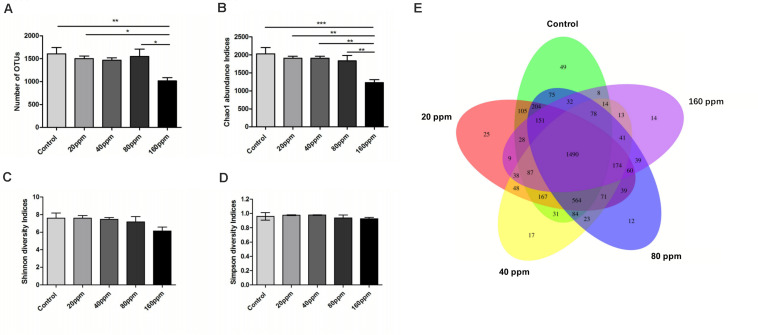
Variations in alpha diversity of the ileum chyme bacteria in Ningxiang pigs. The OTUs level among groups **(A)**. **(B)** Bacterial alpha diversity determined by Chao1 index. **(C)** Bacterial alpha diversity determined by Shannon index. **(D)** Bacterial alpha diversity determined by Simpson index. Each group is represented in a different color. Venn diagrams **(E)** show the numbers of unique and shared OTUs among all groups. *P < 0.05, **P < 0.01, ***P < 0.001 (with FDR adjust).

Figure 1 shows specific bacterial indices (OTUs, Chao1, Shannon, and Simpson) and *P*-values for each group. Significantly higher OTU levels were observed in the control group (*P* < 0.01) and 20 and 80 ppm groups (*P* < 0.05) compared with 160 ppm group (Figure 1A). ε-Polylysine influenced the Chao1 index, which was higher (*P* < 0.05) in the control group than in the treatment groups (Figure 1B); however, no effects were observed on the Shannon and Simpson diversity indices (*P* > 0.05) (Figures 1C,D). The Venn diagrams displayed the unique and shared OTUs in the Ningxiang pigs. Among a total of 3970 OTUs, 37.5% (1490 core OTUs) were shared among the five groups. The numbers of unique OTUs in each group were 49, 25, 17, 12, and 14 (Figure 1E).

**FIGURE 2:**
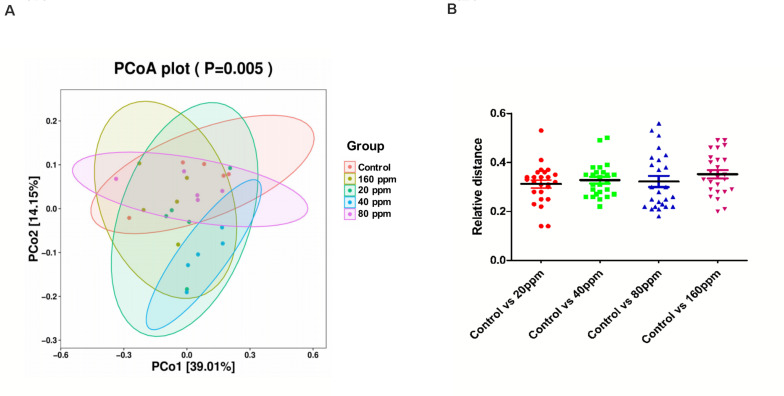
Beta diversity of the ileum chyme bacteria in Ningxiang pigs. PCoA was performed at the operational taxonomic unit (OTU) level based on Bray–Curtis metrics for all samples **(A)** and relative distance of four experimental groups, respectively, compared to the control group **(B)**.

The microbial community structure of all samples was analyzed using the phylogeny-based Bray–Curtis method and visualized using PCoA (Figure 2). The first two factors (PC1 and PC2) accounted for 39.01 and 14.15% of the sample variation, respectively. These results demonstrated that the microbial communities from different groups were distinguishable from one another. The PCoA based on the bacterial OTUs showed that samples clustered together and indicated a shift in the gut bacterial community after ε-polylysine supplementation (Figure 2A). The distance between the experimental groups and control group was 0.33 ± 0.02 (*P* > 0.05) (Figure 2B).

### Effects of ε-Polylysine on the Taxonomic Composition of Gut Bacteria

**FIGURE 3:**
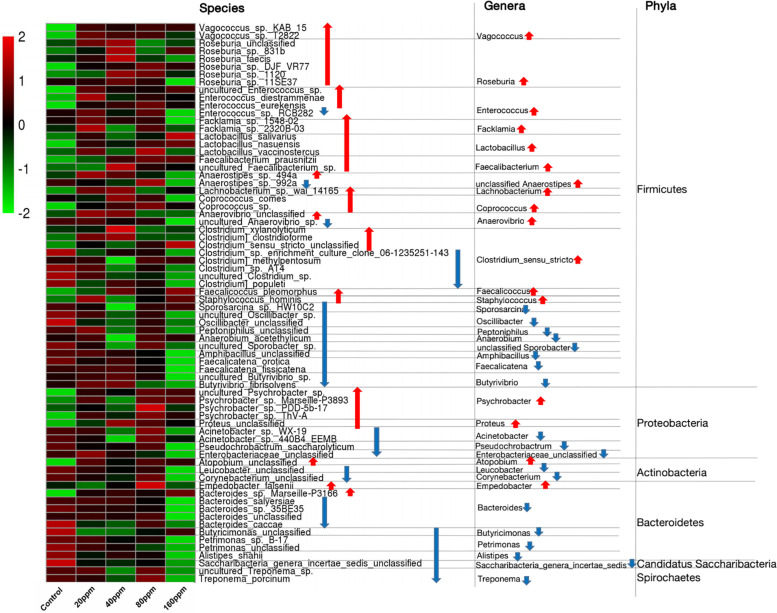
Shifts in the ileum bacterial compositions. Heat map and hierarchical clustering of differentially abundant gut bacterial in piglets with different ε-polylysine supplement level (0, 20, 40, 80, 160 ppm). The values of color in the heat map represent the normalized relative abundances of species (Log 10). Detailed data for heat map were shown in the Supplementary Data 4. Statistics were conducted by ANOVA with Tukey-Kramer test and Benjamini-Hochberg correction among five groups (*n* = 5), and the *P*-value lower than 0.05. Detailed data for the heat map were shown in the Supplementary Data 4.

The ileal microbiotas in the experimental and control groups were examined using the non-parametric Wilcoxon rank-sum test to compare the mean relative abundances of predominant bacteria. Across the samples, 16 different phyla were identified, of which only five phyla had a relative abundance of >1%, namely, Firmicutes (82.35%), Bacteroidetes (7.20%), Proteobacteria (4.23%), Actinobacteria (3.06%), and Fusobacteria (2.00%). The data indicated that the microbial community structure of the ileal contents was similar among the groups at the phylum level. The abundance of Firmicutes significantly increased (*P* < 0.05) with an increase in dietary ε-polylysine. However, the relative abundances of two other phyla (Candidatus Saccharibacteria and Spirochetes) significantly decreased (*P* < 0.05) with an increase in ε-polylysine levels (Supplementary Data 1). The five most abundant phyla accounted for >98.84% of the total sequences in the samples, regardless of the amount of the ε-polylysine supplement. Furthermore, the phyla Proteobacteria, Actinobacteria, and Bacteroidetes exhibited no significant differences (*P* > 0.05). However, certain genera and species within these phyla exhibited significant differences among the groups (*P* < 0.05) (Figure 3).

The taxonomic compositions of the gut bacterial communities were further investigated in the Ningxiang pigs, and a total of 344 genera were identified. Abundant genera were defined as those containing >1% of the total group sequences. The following 23 abundant genera were identified: *Lactobacillus* (9.93%), *Roseburia* (8.19%), *Romboutsia* (6.34%), *Turicibacter* (4.84%), *Clostridium* _XVIII (4.69%), *Lachnospiraceae*_unclassified (4.44%), *Ruminococcaceae*_unclassified (2.89%), *Blautia* (2.69%), *Clostridium*_*sensu*_*stricto* (2.56%), *Terrisporobacter* (2.45%), *Clostridiaceae*_1_unclassified (2.29%), *Eubacterium* (2.00%), *Streptococcus* (2.15%), *Fusobacterium* (1.96%), *Coprococcus* (1.81%), *Oscillibacter* (1.81%), *Clostridium*_XIVa (1.75%), *Bacteroides* (1.56%), *Veillonellaceae*_unclassified (1.27%), *Psychrobacter* (1.01%), *Anaerorhabdus* (1.07%), *Prevotella* (1.08%), and *Phascolarctobacterium* (1.01%). The genus *Lactobacillus* (Firmicutes) was the most abundant in the gut bacterial communities (Supplementary Data 1). Many genera from Firmicutes exhibited an increasing trend (*P* < 0.05), including *Vagococcus*, *Roseburia*, *Enterococcus*, *Facklamia*, *Lactobacillus*, *Faecalibacterium*, unclassified *Anaerostipes*, *Lachnobacterium*, *Coprococcus*, *Anaerovibrio*, *Clostridium*_*sensu*_*stricto*, *Faecalicoccus*, and *Staphylococcus*, while many genera others exhibited a decreasing trend (*P* < 0.05), including *Sporosarcina*, *Oscillibacter*, *Peptoniphilus*, *Anaerobium*, unclassified *Sporobacter*, *Amphibacillus*, *Faecalicatena*, and *Butyrivibrio* (Figure 3 and Supplementary Data 2). Among Proteobacteria, the abundances of the genera *Psychrobacter* and *Proteus* significantly increased (*P* < 0.05), and those of *Acinetobacter*, *Pseudochrobactrum*, and *Enterobacteriaceae*_unclassified significantly decreased (*P* < 0.05). Among Actinobacteria, *Atopobium* exhibited an increasing trend (*P* < 0.05), whereas *Leucobacter* and *Corynebacterium* exhibited a decreasing trend (*P* < 0.05). Furthermore, ε-polylysine significantly increased (*P* < 0.05) *Empedobacter* (Bacteroidetes), *Saccharibacteria*_*genera*_*incertae*_*sedis* (Candidatus_Saccharibacteria), and *Treponema* (Spirochetes), whereas *Bacteroides*, *Butyricimonas*, *Petrimonas*, and *Alistipes* significantly decreased (*P* < 0.05).

We further examined the taxonomic compositions of the ileal microbiotas in the Ningxiang pigs at the species level. In total, 672 significant species were identified. The 20 most abundant species included *Lactobacillus amylovorus*, *Romboutsia*, *Turicibacter* sp., *Clostridium*_XVIII, *Lachnospiraceae*, *Ruminococcaceae*, *Roseburia* sp., *Clostridium*_*sensu*_*stricto*, *Terrisporobacter*, *Clostridiaceae*_1, *Roseburia*, *Roseburia* sp. 831b, *Eubacterium* sp., *Clostridium*_XlVa, *Coprococcus* sp., *Oscillibacter*, *Fusobacterium* sp., *Blautia*_sp., *Veillonellaceae*, and *Anaerorhabdus* sp. Dietary ε-polylysine significantly increased or decreased (*P* < 0.05) the abundances of several species (Figure 3 and Supplementary Data 3, 4). The relative abundances (>0.05%) of 16 species, including *Roseburia* sp. 831b, *Roseburia faecis*, *Facklamia* sp. 2320B-03, and *Coprococcus* sp. increased, whereas those of 11 species, including *Acinetobacter* sp. WX-19, *Bacteroides salyersiae*, and *Bacteroides caccae* decreased. Most of these species belong to the phylum Firmicutes (Supplementary Data 4).

### Prediction of Ileal Microbiota Function in Ningxiang Pigs

**FIGURE 4:**
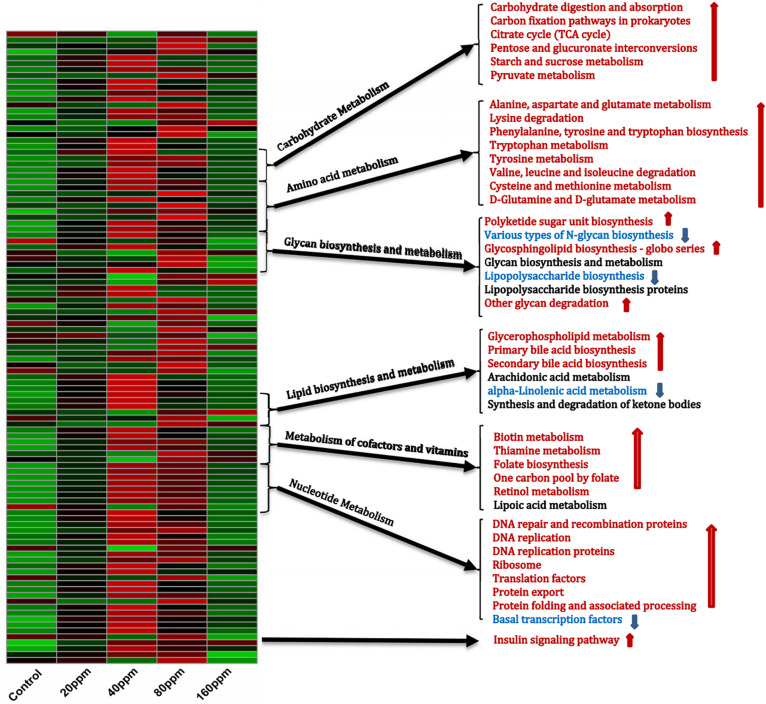
Shifts in ileum bacterial functional profiles as ε-polylysine supplement level. Heat map and hierarchical clustering of different abundant KEGG pathways identified at different ε-polylysine supplement level (0, 20, 40, 80, 160 ppm). The values of color in the heat map represent the normalized relative abundance of KEGG pathways (Log 10). Detailed data for the heat map were shown in the Supplementary Data 5.

The PICRUSt analysis, which was used to investigate the functional profiles of the microbiota, suggested distinct nutrient source utilization patterns in the ileum, depending on the bacterial composition (Figure 4). Dietary ε-polylysine supplementation resulted in a significant increase in metabolic activity (*P* < 0.05), as the abundances of genes involved in carbohydrate, amino acid, and fatty acid metabolism increased. The bacterial community exhibited significant increases (*P* < 0.05) in the relative abundances of genes for carbohydrate digestion and absorption, carbon fixation pathways in prokaryotes, tricarboxylic acid cycle, pentose and glucuronate interconversions, and starch, sucrose, and pyruvate metabolism. Furthermore, the relative abundances of genes involved in the metabolism of alanine, aspartate, glutamate, tryptophan, tyrosine, cysteine, methionine, D-glutamine, and D-glutamate, as well as in the degradation of lysine, valine, leucine, and isoleucine and in the biosynthesis of phenylalanine, tyrosine, and tryptophan, were also predicted to increase. The relative abundances of genes involved in the biosynthesis and metabolism of glycan varied. Dietary ε-polylysine significantly increased (*P* < 0.05) the proportions of genes for polyketide sugar unit biosynthesis, glycosphingolipid biosynthesis–globo series, and other glycan degradation significantly decreased (*P* < 0.05) the proportions of genes for *N*-glycan and lipopolysaccharide biosynthesis. Furthermore, fatty acid biosynthesis and metabolism were affected by the increased proportions of genes for glycerophospholipid metabolism and primary and secondary bile acid biosynthesis, as well as by the decreased proportions of genes for alpha-linolenic acid metabolism. Additionally, the bacterial community exhibited significantly increased relative abundances of genes for biotin, thiamine, and retinol metabolism, folate biosynthesis, and the folate-dependent one-carbon pool (Figure 4), which are all important for maintaining normal physiological functions in animals. The results also showed increases in the proportions of genes involved in molecular processes essential for cell functions and maintenance, such as DNA repair and recombination proteins, DNA replication, DNA replication proteins, ribosomes, translation factors, protein export, protein folding, and associated processing, whereas basal transcription factors were decreased. Finally, the bacterial community exhibited increased proportions of genes for the insulin signaling pathway (Supplementary Data 5).

### Correlation Between Intestinal Microorganisms and Utilization of Nutrients

At the phylum level, the abundances of Firmicutes and Candidatus Saccharibacteria were positively correlated with CP digestibility (*P* < 0.05), while that of Proteobacteria was negatively correlated with CP digestibility (*P* < 0.05). The abundance of Candidatus Saccharibacteria was positively correlated with the efficiency of ME utilization and CF digestibility (*P* < 0.01). At the genus level, the abundances of *Guggenheimella* and *Saccharibacteria_genera_incertae_sedis* were positively correlated with CP digestibility (*P* < 0.05). However, the abundances of *Phascolarctobacterium*, *Coprococcus*, *Faecalicoccus*, *Pseudoflavonifractor*, *Fretibacterium*, *Ignatzschineria*, and *Bittarella* were negatively correlated with CP digestibility (*P* < 0.05). The abundances of *Guggenheimella*, *Saccharibacteria_incertae*_*sedis*, *Leucobacter*, *Petrimonas*, *Jeotgalicoccus*, and *Halomonas* were positively correlated with the efficiency of ME utilization and CF digestibility (*P* < 0.05), but those of *Phascolarctobacterium*, *Coprococcus*, *Faecalicoccus*, *Pseudoflavonifractor*, *Fretibacterium*, and *Faecalibacterium* were negatively correlated with the efficiency of ME utilization and CF digestibility (*P* < 0.05). In addition, the abundances of *Blautia* and *Ignatzschineria* were negatively correlated with the efficiency of ME utilization (*P* < 0.05) (Table 4).

**TABLE 4:** Intestinal microorganisms correlated to the utilization of nutrients and Pearson’s correlation between gut microbiota and utilization of nutrients.

Items	CP digestibility Pearson’s correlation	ME utilization Pearson’s correlation	CF digestibility Pearson’s correlation
**Phyla**			
Firmicutes	0.4262*	0.1842	0.1644
Candidatus Saccharibacteria	0.4682*	0.5200**	0.5086**
Proteobacteria	−0.5106**	–0.1535	–0.0079
**Genera**			
*Guggenheimella*	0.3980**	0.5172*	0.5702**
*Saccharibacteria_ genera_ incertae_ sedis*	0.4682*	0.5200**	0.5086**
*Leucobacter*	0.2566	0.4848*	0.5342**
*Petrimonas*	0.3872	0.4870*	0.5156**
*Jeotgalicoccus*	0.1413	0.4939*	0.5454**
*Halomonas*	0.2341	0.4048*	0.5140**
*Phascolarctobacterium*	−0.7221**	−0.6130**	0.5143**
*Coprococcus*	−0.4568*	−0.5385**	−0.4939*
*Faecalicoccus*	−0.4316*	−0.4588*	−0.4660*
*Pseudoflavonifractor*	−0.5376**	−0.4557*	−0.4585*
*Fretibacterium*	−0.4468*	−0.5038*	−0.4687*
*Faecalibacterium*	–0.3221	−0.4007*	−0.4867*
*Ignatzschineria*	−0.7358**	−0.5266**	–0.3427
*Bittarella*	−0.5637**	−0.4677*	–0.3113

* The correlation is significant at a level of 0.05; ** the correlation is significant at a level of 0.01.

## Discussion

To date, no study has investigated the effects of ε-polylysine on digestibility in pigs. However, previous studies investigated the effects of dietary lysine on digestibly and found that lysine deficiency or excess could influence nutrient digestibility in pigs (Kim et al., 2011; Zeng et al., 2013; Elsbernd et al., 2017), which can affect nitrogen retention and whole-body protein turnover (Roy et al., 2000; Yin et al., 2018). In the current study, ε-polylysine showed some effects on CP and CF digestibility and ME utilization in Ningxiang pigs. A previous study showed that changes in the abundances of bacterial genera were correlated with apparent CF digestibility and the abundance of *Clostridium* was associated with dietary fiber metabolism (Niu et al., 2015). Our findings were also similar to those of previous studies, wherein dietary supplementation with amino acids was reported to mediate the gut microbiota composition and diversity, which may further affect the host metabolism and health (Ren et al., 2014; Bin et al., 2017; Ji et al., 2018). Jumpertz et al. (2011) showed that nutrients could change the gut (fecal) bacterial community structure over a short period of time, and the observed associations between gut microbes and nutrient absorption indicated a possible role of the human gut microbiota in the regulation of the nutrient balance.

Therefore, we investigated the alterations in the ileal microbiota of Ningxiang pigs that were fed a diet supplemented with different concentrations of ε-polylysine. Our results demonstrated alterations in the gut microbiota composition at the phylum, genus, and species levels. Consistent with the data of previous studies (Chen et al., 2018; Wang et al., 2019), Firmicutes, Bacteroidetes, and Proteobacteria were the three most dominant phyla in the gut microbiota of pigs, of which the most abundant phylum was Firmicutes, followed by Bacteroidetes and Proteobacteria. Our findings were also similar to those of Lee et al. (2018), who reported that the abundance of Spirochetes in pigs was decreased by the supplementation of plant extracts. Wang et al. (2018) demonstrated that diabetic cognitive dysfunction in mice was associated with increased production of bile acids in the liver and activation of bile acid signaling in the intestine. Moreover, the bacterial community composition was altered in the cecum of these mice and was characterized by a marked increase in the population of Candidatus Saccharibacteria. Because the abundance of Candidatus Saccharibacteria was decreased by ε-polylysine in the present study, its action may be associated with the suppression of bile secretion and primary and secondary bile acid biosynthesis. However, this association should be elucidated in further studies.

At the genus level, *Lactobacillus*, including *L. salivarius*, *L. nasuensis*, and *L. vaccinostercus*, has been demonstrated to be essential for improving the intestinal microbial balance (Zhang et al., 2019). In addition, *L. salivarius* has probiotic properties; it activates a broad range of cytokines and chemokines and elicits immunomodulatory activity by enhancing innate and acquired immune responses (Perez-Cano et al., 2010; Zhang et al., 2011; Larsen et al., 2013). *Roseburia*, which is a core genus in representative populations of the world, along with *Faecalibacterium*, *Eubacterium*, *Clostridium*, *Blautia*, and *Ruminococcus* (Dehingia et al., 2015), was the second most predominant genus in this study. Five species of *Roseburia*, including *R. faecis*, *Roseburia* sp. 831b, *Roseburia* sp. DJF VR77, *Roseburia* sp. 1120, and *Roseburia* sp. 11SE37, were increased by ε-polylysine. Gut *Roseburia* spp. metabolized dietary components, which stimulated their proliferation and metabolic activities (Supplementary Data 3, 4). Tamanai-Shacoori et al. (2017) reported that the genus *Roseburia* included commensal bacteria that produce short-chain fatty acids (SCFAs), especially butyrate, which affects colonic motility, immunity maintenance, and anti-inflammatory properties. In this study, the increase in the *Roseburia* abundance by ε-polylysine may have induced SCFA production and improved gut immunity. Furthermore, PICRUSt analysis suggested that ε-polylysine exerted strong effects on carbohydrate metabolism, glycan biosynthesis, and other metabolic functions, which may be related to the presence of bacteria of the genera *Vagococcus*, *Enterococcus*, *Facklamia*, *Rothia*, *Lachnobacterium*, *Proteus*, *Coprococcus*, *Atopobium*, *Anaerovibrio, Anaerorhabdus*, and *Staphylococcus*. Butyrate, a fermentation product of these bacteria can decompose glycan and carbohydrates, and also affects the biosynthesis and metabolism of fatty acids (Leonel and Alvarez-Leite, 2012). However, ε-polylysine could also exhibit inhibitory effects on the biosynthesis and metabolism of carbohydrates, glycan, and fatty acids by inducing changes in the abundances of some other genera (*Butyricimonas*, *Leucobacter*, *Atopostipes*, *Amphibacillus*, *Alloiococcus*, *Alistipes*, and *Butyrivibrio*). Furthermore, ε-polylysine increased the relative abundances of *Faecalibacterium* and *Clostridium*_*sensu*_*stricto*, which are beneficial bacteria that can inhibit pathogens. *Faecalibacterium* spp. can promote the development and proliferation of probiotics, such as *Bifidobacterium* spp. and lactic acid bacteria (Duncan et al., 2002; Yu et al., 2019). In addition, ε-polylysine inhibited some pathogenic bacteria (*Micrococcaceae* unclassified, *Acinetobacter*, *Anaerococcus*, *Peptoniphilus*, *Dehalobacterium*, *Finegoldia*, *Treponema*, and *Brevundimonas*). Moreover, Krizova et al. (2002) investigated enzymes of the butyrate pathway and fermentation patterns and reported that *Coprococcus* sp. from the human gut, which produces high levels of butyric acid *in vitro* and is a net producer of acetate, had detectable butyrate kinase, acetate kinase, and butyryl-CoA:acetate-CoA transferase activities. Butyric acid is an important SCFA, and Oh et al. (2019) demonstrated that SCFAs can activate the AMPK/PPAR pathway directly or by activating adipose tissue and can ultimately regulate fatty acid oxidation. Thus, *Coprococcus* sp. could be associated with fatty acid biosynthesis and metabolism in the present study.

Consistent with the data of a previous study on gut microbiome in pigs (Yang and Liao, 2019), the results of our study suggested an increase in the metabolism of carbohydrates, amino acids, cofactors, and vitamins. Furthermore, significant increases in almost all KEGG pathways were associated with nucleotide metabolism, except for basal transcription factors. A remarkable increase in the activity of the insulin signaling pathway was also predicted. Regarding fatty acid biosynthesis and metabolism, the predicted changes included an increase in the glycerophospholipid metabolism and biosynthesis of primary and secondary bile acids, as well as a decrease in the alpha-linolenic acid metabolism. Primary bile acids are produced by the liver to dissolve dietary lipids and fat-soluble vitamins in the small intestine. The primary bile acid pool mainly circulates back to the liver; however, a small portion of the bile acid pool (approximately 5%) enters the large intestine and is further metabolized into secondary bile acids by intestinal microorganisms (Chiang, 2009). The two main bile acid receptors that regulate the host metabolism are G-protein-coupled bile acid receptor 1 and the farnesol X receptor, which, together with bile acids and the intestinal microbiome, regulate the synthesis, metabolism, and distribution of bile acids *in vivo* (Molinaro et al., 2018). Jia et al. (2018) reported that bile acids played an important role in the lipid balance, carbohydrate metabolism, insulin sensitivity, and innate immunity. In addition, ε-polylysine exerted complex effects on glycan biosynthesis and metabolism, thereby regulating the body energy balance via changes in the gut microbiota in Ningxiang pigs.

This study revealed changes in nutrient digestibility caused by different ε-polylysine levels and the relationships between the abundance and diversity of the gut microbiota in Ningxiang pigs. Jumpertz et al. (2011) indicated a possible role of the gut microbiota in the regulation of nutrient absorption in humans. In a previous study (Ashida et al., 2012), the gut microbiota was shown to degrade dietary fiber, and the abundances of bacteria belonging to the genera *Guggenheimella*, *Saccharibacteria incertae sedis*, *Leucobacter*, *Petrimonas*, *Jeotgalicoccus*, and *Halomonas* were positively correlated with CF digestibility. Jha and Berrocoso (2015) reported that dietary fiber negatively affected energy and nutrient digestibility, which may indicate a balance between CP and CF digestibility. The relative abundances of nine genera, namely, *Guggenheimella*, *Saccharibacteria incertae sedis*, *Phascolarctobacterium*, *Coprococcus*, *Faecalicoccus*, *Pseudoflavonifractor*, *Fretibacterium*, *Ignatzschineria*, and *Bittarella*, were correlated with CP digestibility. The gut microbiota plays a key role in controlling the energy balance via energy expenditure and storage (Ridaura et al., 2013). Foditsch et al. (2014) suggested that *Faecalibacterium prausnitzii* was related to the energy-harvesting capacity of the intestinal microbiota, which is consistent with our results. Therefore, the relationship between nutrient (CP, ME, and CF) digestibility and the gut microbiota should be the focus of further study.

In conclusion, considering that there were no significant differences in ADFI, the reduced nutrient digestibility led to an increased excretion of nutrients and increased metabolism of carbohydrates, amino acids, fatty acids, and glycan, which may signify a reduced nutritional requirement for Ningxiang pigs. We determined the structure and function of the microbiota in the ileal contents of Ningxiang pigs, which were fed dietary ε-polylysine, and predicted that ε-polylysine could enhance the level of nutrients, including carbohydrates, vitamins, and glycan, thus providing health benefits to pigs.

## Data Availability Statement

The datasets presented in this study can be found in online repositories. The names of the repository/repositories and accession number(s) can be found in the article/Supplementary Material.

## Ethics Statement

The animal study was reviewed and approved by the Institute of Bast Fiber Crops, Chinese Academy of Agricultural Sciences.

## Author Contributions

XZ, DW, QD, and X Yan designed the research. XZ and X Yu conducted the research. XZ, BX, CX, and ZW analyzed the data. XZ and DW wrote the manuscript. All authors read and approved the final version of the manuscript.

## Conflict of Interest

The authors declare that the research was conducted in the absence of any commercial or financial relationships that could be construed as a potential conflict of interest. The reviewer XK declared a shared affiliation with several of the authors, XZ, XY, ZH, DW, QD, to the handling editor at time of review.

## Supplementary Material

Supplementary Figure S1 The length distribution of sequences.

Supplementary Figure S2 Bacterial rarefaction curves based on good_coverage was used to assess the depth of coverage for each sample. Each sample was distinguished by different colors of lines.
