# Supplementary figures and images for: Dietary Supplementation of ε-Polylysine Beneficially Affects Ileal Microbiota Structure and Function in Ningxiang Pigs
Source: Front Microbiol. 2020 Nov 16;11:544097. doi: 10.3389/fmicb.2020.544097 (PMC7702972; doi:10.3389/fmicb.2020.544097)

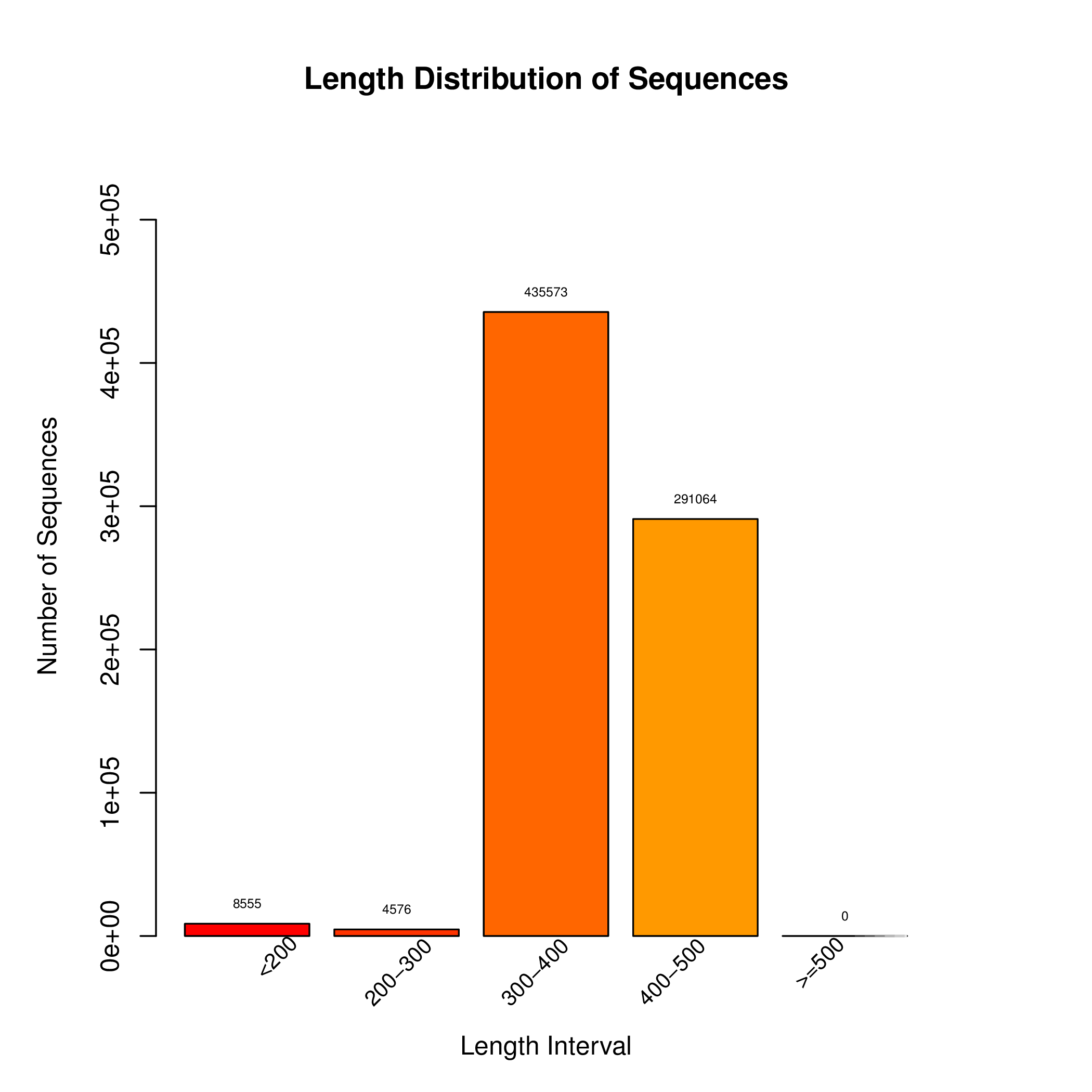

Supplement: Supplementary Figure S1 — The length distribution of sequences. [file Image_1.tiff]

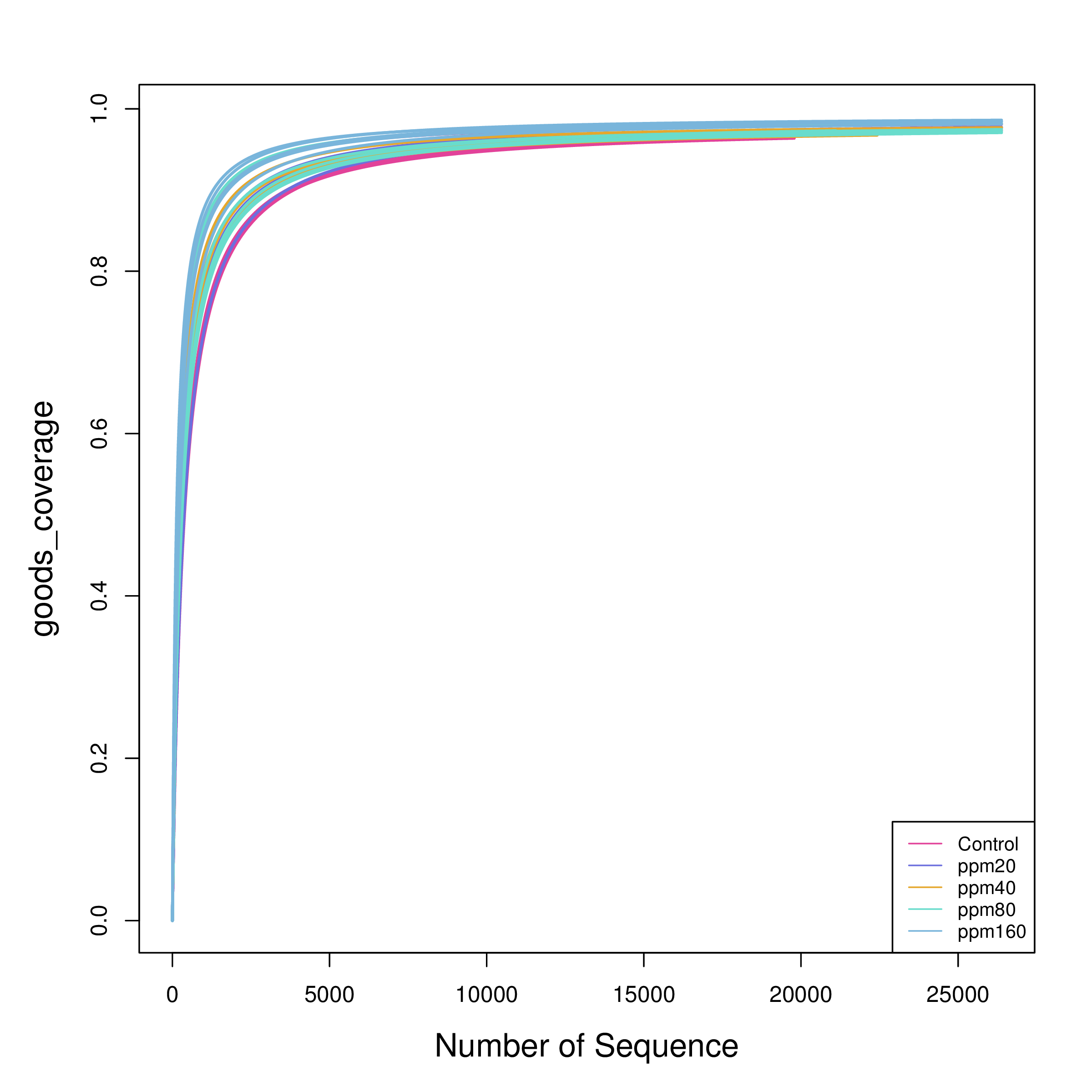

Supplement: Supplementary Figure S2 — Bacterial rarefaction curves based on good_coverage was used to assess the depth of coverage for each sample. Each sample was distinguished by different colors of lines. [file Image_2.tiff]
